# Green analytical method for the simultaneous analysis of cytochrome P450 probe substrates by poly(*N*-isopropylacrylamide)-based temperature-responsive chromatography

**DOI:** 10.1038/s41598-020-65270-z

**Published:** 2020-06-01

**Authors:** Yutaro Maekawa, Naoya Okamoto, Yuji Okada, Kenichi Nagase, Hideko Kanazawa

**Affiliations:** Faculty of Pharmacy, Keio University, 1-5-30, Shibakoen, Minato-ku Tokyo, 105-8512 Japan

**Keywords:** Analytical chemistry, Green chemistry

## Abstract

High-performance liquid chromatography (HPLC) is the most common analytical method practiced in various fields and used for analysis of almost all drug compounds in the pharmaceutical industries. During drug development, an evaluation of potential drug interaction with cytochrome P450 (CYP) is essential. A “cocktail” approach is often used in drug development to evaluate the effect of a drug candidate on multiple CYP enzymes in a single experiment. So far, simultaneous analysis of multiple CYP substrates, which have greatly different structure and physicochemical properties, has required organic solvents and mobile phase gradient methods. However, despite the recent emphasis on environmental protection, analytical methods that use only aqueous solvents without the use of organic solvents for separation have not been studied well. This study sought to develop the simultaneous analysis of multiple CYP substrates by using poly(*N*-isopropylacrylamide) (PNIPAAm)-based temperature-responsive chromatography with only aqueous solvents and isocratic methods. Good separation of multiple CYP substrates was achieved without using organic solvents and any gradient methods by temperature-responsive chromatography utilizing a P(NIPAAm-*co*-*n*-butyl methacrylate (BMA))- and P(NIPAAm-*co*-*N*-acryloyl L-tryptophan methyl ester (L-Trp-OMe))-grafted silica column. Overall, PNIPAAm-based temperature-responsive chromatography represents a remarkably simple, versatile, and environmentally friendly bioanalytical method for CYP substrates and their metabolites.

## Introduction

Since Anastas and Warner outlined the twelve principles of green chemistry over two decades ago^1^, green chemistry has become a standard concept among scientists in the pharmaceutical and chemical industries as well as academia. As noted during the United Nations Conference on Climate Change held in France in 2015 (COP21^2^), the necessity to limit the accumulation of pollution sources and waste has reached a critical level^3^ and the health of consumers and the environment has become increasingly important^4^. High-performance liquid chromatography (HPLC) has been utilized extensively for research, usually to determine the identity of organic compounds. However, HPLC analysis can be wasteful; indeed, a single analysis can produce over 1 L of liquid waste each day^5,6^. To minimize such waste, green chromatography has been developed as an effective analytical method^5,6^, particularly because the most common HPLC method, reversed-phase liquid chromatography (RPLC), suffers from a variety of drawbacks such as the requirement of large volumes of organic solvents for the mobile phase, column cleaning, and a lengthy process for determining optimal mobile phase and gradient methods.

The interaction between drugs, which is known as drug–drug interactions, refer to the ability of a drug or its metabolites to alter the pharmacokinetics, such as absorption, distribution, metabolism, and/or elimination of other drugs or their metabolites. Almost all drug–drug interactions occur during drug metabolic processes facilitated by cytochrome P450 (CYP)^7–9^. CYP have many isoforms and their substrate specificity is low since there are many substrates having greatly different structure and physicochemical properties such as hydrophobicity. Assessing the potential effects of a drug on CYP enzymes at an early round of drug development is an important aspect of proper risk assessment of new drug candidates for pharmaceutical industries so that they can decide whether to design a robust clinical development strategy or to abandon such efforts^10–12^.

A “cocktail” approach is often used in drug development for *in vitro* and clinical drug interaction studies to evaluate the effect of a drug candidate on multiple CYP enzymes in a single experiment. In this way, alteration of concentration of each CYP enzyme substrate with and without the drug candidate present can be systematically evaluated. Such an approach could contribute to cost and time savings to pharmaceutical industries while screening drug candidates or assessing their potential in humans because it would reduce the number of *in vitro* and clinical drug interaction studies. Although some studies have reported a simultaneous analytical method for multiple CYP substrates using organic solvents and mobile phase gradient methods^13–15^, until now, there have been no reports of a simultaneous analytical method that is performed with only aqueous solvents and isocratic methods.

Poly(*N*-isopropylacrylamide) (PNIPAAm) is referred to the most reported temperature-responsive polymers and shows a rapid phase transition between hydrophobic and hydrophilic in aqueous solutions at the lower critical solution temperature (LCST) near 32 °C^16,17^. The PNIPAAm chains are dehydrated and hydrophobic above the LCST, whereas hydrated below this temperature to become hydrophilic^18–20^. Plus, the characteristics of PNIPAAm can be grafted by introducing various *co*-monomers into the polymer unit^21–23^. As the illustration, *n*-butyl methacrylate (BMA) as a *co*-monomer leads to increased hydrophobicity, and the use of an aromatic amino acid *co*-monomer, such as *N*-acryloyl L-phenylalanine methyl ester (L-Phe-OMe), leads to enhanced molecular recognition^21–23^. By utilizing these characteristics, temperature-responsive chromatography with PNIPAAm-grafted stationary phase has been developed^24^. Interestingly, while it is impossible for a conventional RPLC to alter the properties of its stationary phase, this PNIPAAm-based chromatography can drastically and reversibly alter the stationary phase properties with only aqueous mobile phase by changing only temperature. In other words, this chromatography can control a separation selectivity by changing temperature, unlike RPLC, which requires a mobile phase gradient method using organic solvents to enhance its separation selectivity^24,25^. In addition, this column can be cleaned with only cold water while organic solvents are used for conventional RPLC. That is, temperature-responsive chromatography with PNIPAAm-grafted stationary phase is simpler and more environmentally friendly than RPLC. This unique temperature-responsive property of PNIPAAm has been widely applied in drug delivery systems^26–31^, enzyme bioconjugates^32^, microfluidics^33,34^, fluorescent polymer probes^35–37^, and materials for regenerative medicine^38–43^. So far, simultaneous analysis by this temperature-responsive chromatography has been reported for analytes having similar chemical structures such as barbiturates and benzodiazepines^23^. However, no simultaneous analysis using this technique of multiple analytes with greatly different structure and physicochemical properties has been reported.

The current study sought to develop the simultaneous analytical method of multiple CYP substrates by using PNIPAAm-based temperature-responsive chromatography with only aqueous solvents and isocratic methods. In this study, three different experiments using PNIPAAm-based temperature-responsive chromatography were conducted to demonstrate the simultaneous analysis of: (1) a cocktail of multiple CYP substrates for *in vitro* drug interaction assays, (2) CYP substrates and their metabolites, and (3) a cocktail of multiple CYP substrates for clinical drug interaction studies. This PNIPAAm-based temperature-responsive chromatography represents a remarkably simple and environmentally friendly analytical method.

## Results and discussion

### Characteristics of PNIPAAm-based polymers

**Figure 1:**
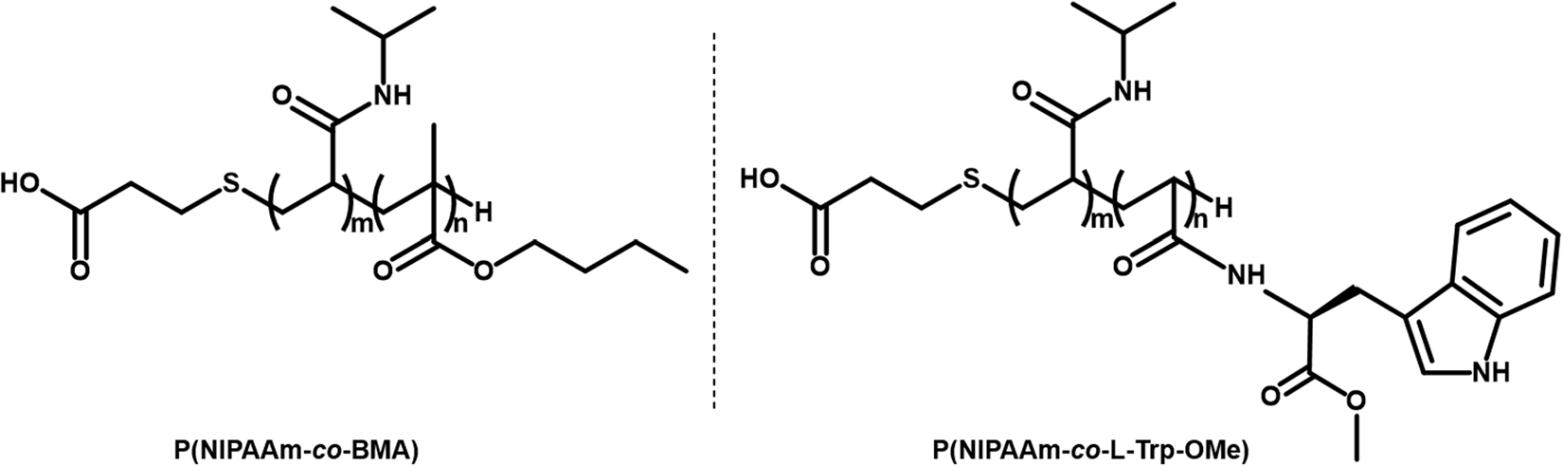
Structures of PNIPAAm-based temperature-responsive polymers used in this study.

**Figure 2:**
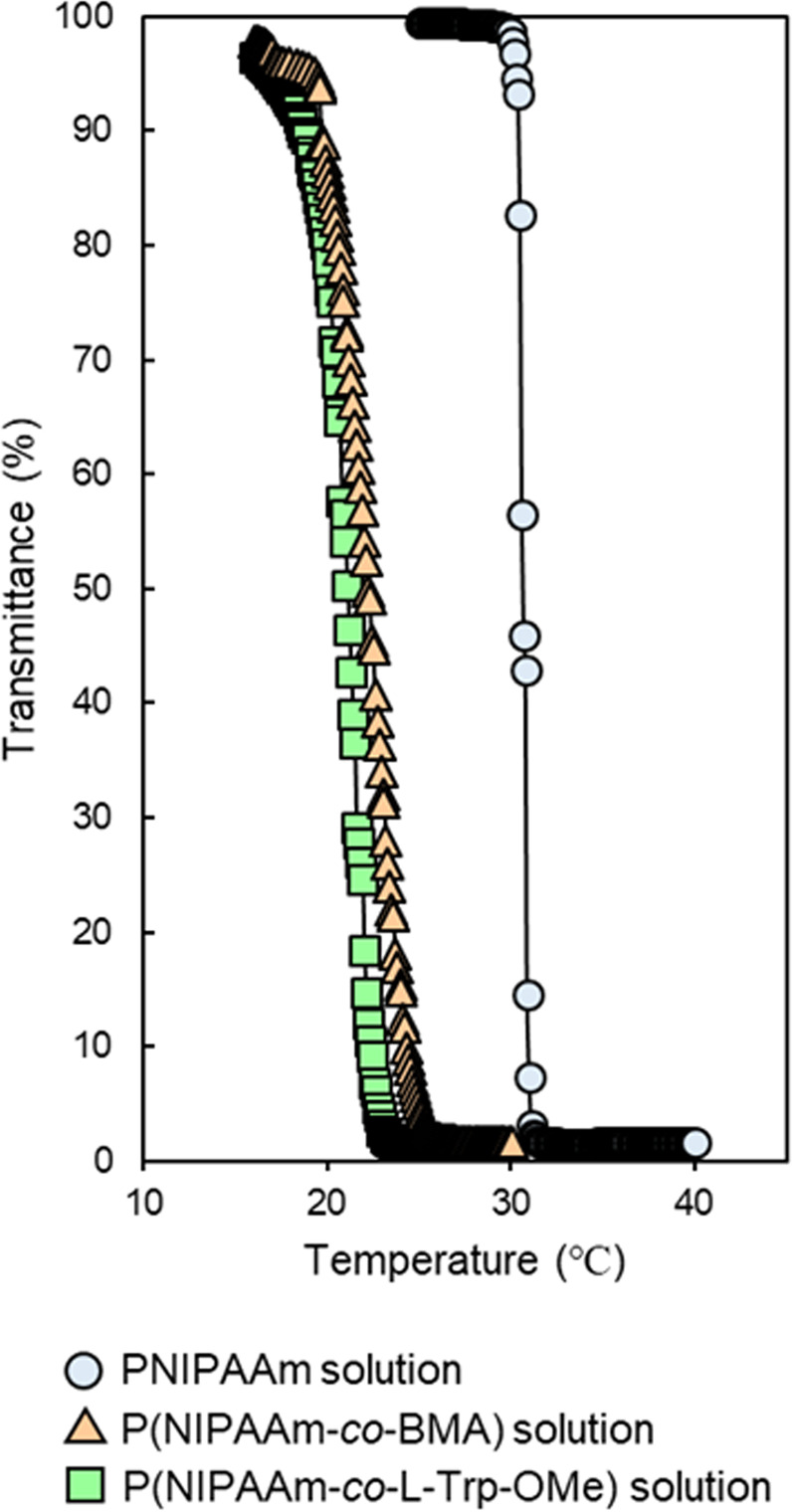
Temperature dependence of transmittance of 5 mg mL^−1^ PNIPAAm solution, P(NIPAAm-*co*-BMA) solution, and P(NIPAAm-*co*-L-Trp-OMe) solution.

P(NIPAAm-*co*-BMA) and P(NIPAAm-*co*-*N*-acryloyl L-tryptophan methyl ester (L-Trp-OMe)) (Fig. 1) were synthesized from NIPAAm with BMA and L-Trp-OMe through radical polymerization using 3-mercaptopropionic acid (MPA). The LCSTs of P(NIPAAm-*co*-BMA) and P(NIPAAm-*co*-L-Trp-OMe) were 22.1 and 21.4 °C, respectively (Fig. 2). These LCSTs corresponded well with those reported previously^44,45^. The LCST can be changed depending on the hydrophobicity/hydrophilicity of the monomer^36^. BMA and L-Trp-OMe would be expected to have similar hydrophobicity. As with PNIPAAm, the sharp transitions were observed in P(NIPAAm-*co*-BMA) and P(NIPAAm-*co*-L-Trp-OMe) at the LCST (Fig. 2), suggesting that the silica beads grafted with these polymers rapidly change the surface physicochemical properties at each LCST.

### Simultaneous analysis of six CYP substrates for *in vitro* drug interaction assays

**Figure 3:**
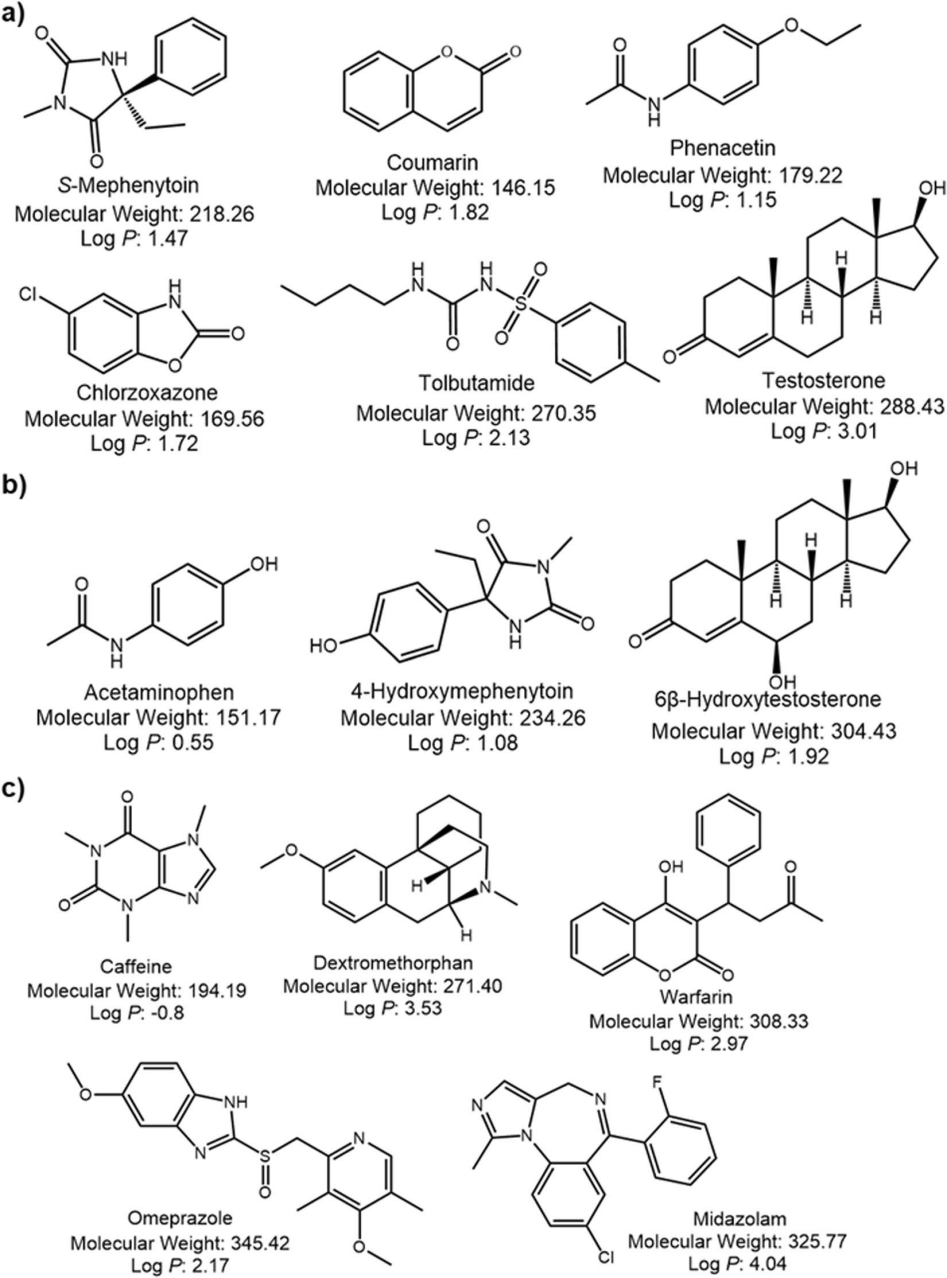
Structures of the analytes used in simultaneous analysis of six CYP substrates for *in vitro* drug interaction assay (**a**), separation of CYP substrates and metabolites by temperature-responsive chromatography (**b**), and simultaneous analysis of five CYP substrates for clinical drug interaction studies (**c**).

**Figure 4:**
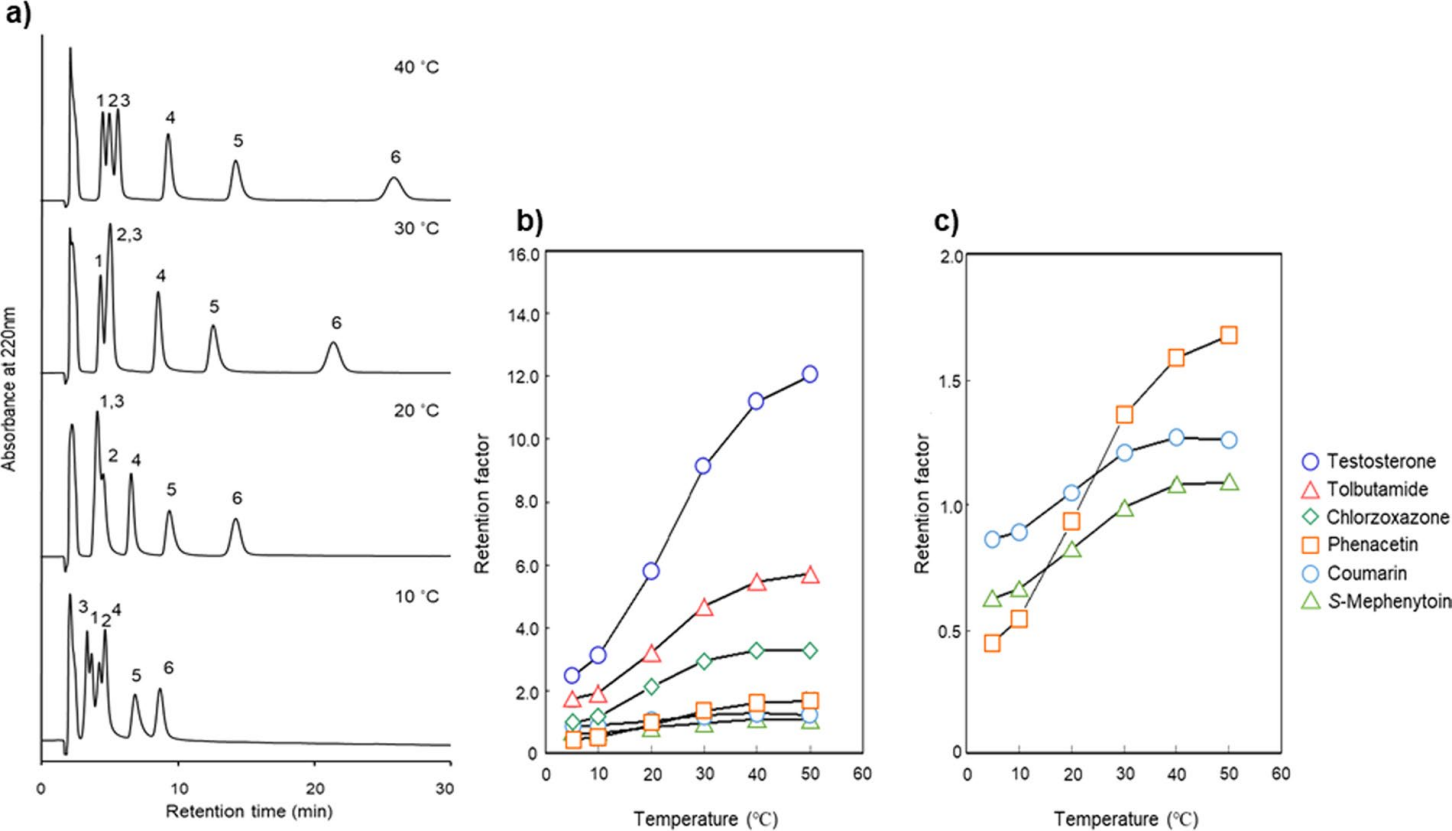
Chromatogram and retention factor of six CYP substrates (1: *S*-Mephenytoin, 2: Coumarin, 3: Phenacetin, 4: Chlorzoxazone, 5: Tolbutamide, 6: Testosterone) at various column temperatures on P(NIPAAm-*co*-BMA) column (**a**); effect of temperature on the retention factor (**b**); enlargement of (**b**) for *S*-Mephenytoin, coumarin, and phenacetin (**c**).

**Figure 5:**
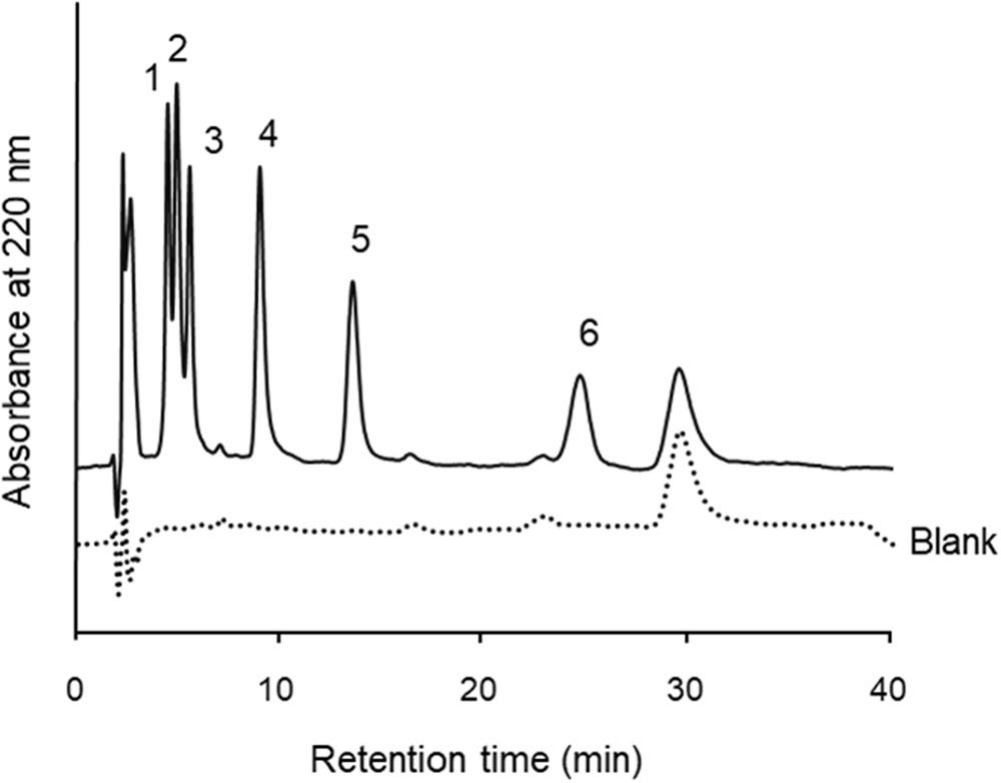
Chromatogram of six CYP substrates (1: *S*-Mephenytoin, 2: Coumarin, 3: Phenacetin, 4: Chlorzoxazone, 5: Tolbutamide, 6: Testosterone) spiked in serum.

Six CYP substrates (phenacetin for CYP1A2, coumarin for CYP2A6, tolbutamide for CYP2C9, *S*-mephenytoin for CYP2C19, chlorzoxazone for CYP2E1, and testosterone for CYP3A4; Fig. 3a) were chosen from CYP substrates identified in the drug interaction guide provided by the U.S. Food and Drug Administration (FDA)^46^ and commonly used for *in vitro* drug interaction assays^47,48^. These six drugs were analyzed using the P(NIPAAm-*co*-BMA) column at various column temperatures with an isocratic aqueous mobile phase, 0.1 M ammonium acetate buffer at pH 4.8, and almost separated at 40 °C (Fig. 4a). The retention factors of these substrates increased as the temperature increased (Fig. 4b,c). Although *S*-mephenytoin, coumarin, and phenacetin were only separated at the peak tips, these 3 analytes could be better separated by a longer column. Interestingly, the elution order of phenacetin, *S*-mephenytoin and coumarin changed at column temperatures above the LCST (Fig. 4c). This alteration likely results from the changing affinity of phenacetin with the stationary phase as increasing column temperature. Though, the hydrophilicity of phenacetin was the highest of the six CYP substrates examined. Other factors such as interaction between polymer chain and the NH group of phenacetin would be involved as previously reported^21–23^. Besides, after spiking the six analytes in human blood serum, the corresponding peaks in the chromatogram were well separated without overlapping impurities under the same HPLC conditions at a column temperature of 40 °C (Fig. 5). The elution order of the spiked analytes was similar to their intrinsic profiles. This experiment suggests that by using a P(NIPAAm-*co*-BMA) column, the cocktail of CYP substrates for *in vitro* evaluation^46–48^ in biological sample can be simultaneously analyzed without overlapping impurities from biological fluid.

### Separation of CYP substrates and metabolites by temperature-responsive chromatography

**Figure 6:**
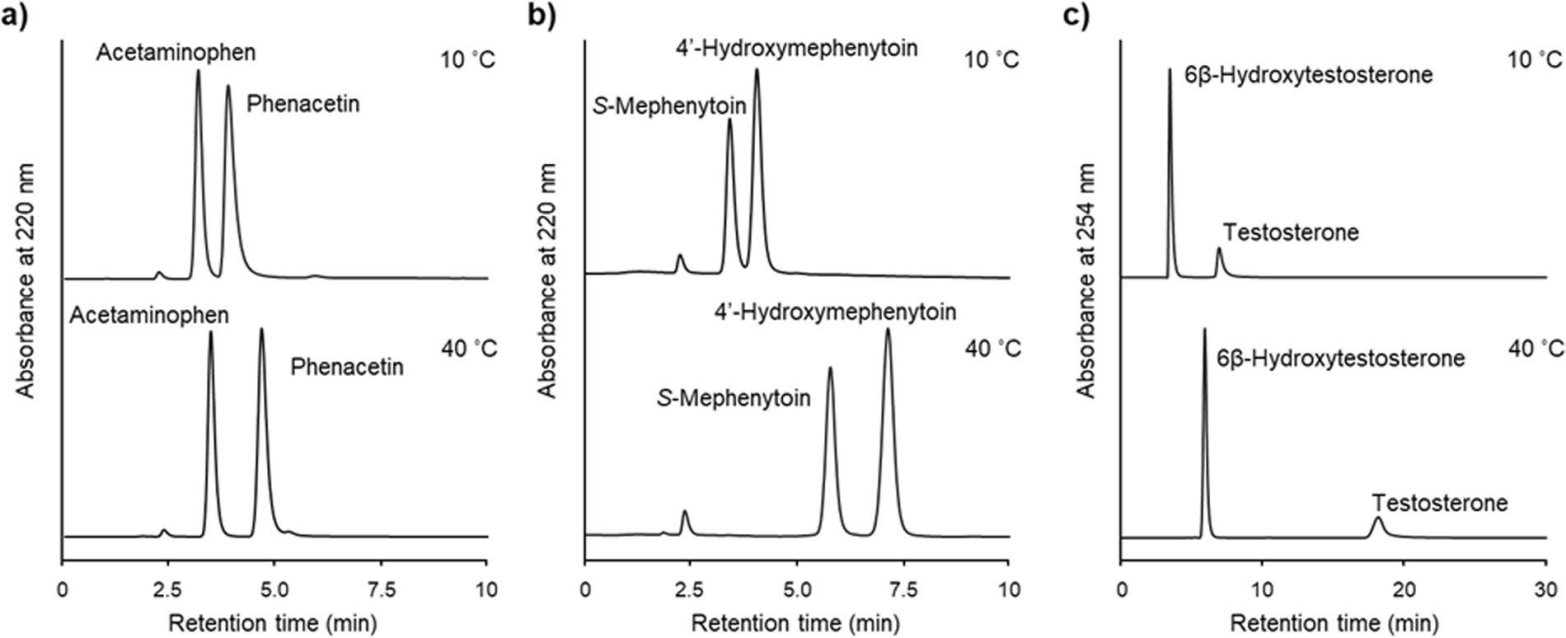
Effect of temperature on the retention times of CYP substrate and its metabolite at 10 °C and 40 °C (phenacetin/acetaminophen (**a**); *S*-mephenytoin/4′-hydroxymephenytoin (**b**); testosterone/6β-hydroxytestosterone (**c**)).

Three mixtures of CYP substrates and their metabolites (phenacetin/acetaminophen, *S*-mephenytoin/4′-hydroxymephenytoin, and testosterone/6β-hydroxytestosterone; Fig. 3b) were analyzed using the P(NIPAAm-*co*-BMA) column with an isocratic aqueous mobile phase, 0.1 M ammonium acetate buffer at pH 4.8 or deionized water, at column temperatures of 10 °C and 40 °C, respectively (Fig. 6). As the column temperature was increased, the retention times of these substrates and their metabolites were delayed. The elution orders of phenacetin/acetaminophen (Fig. 6a) and testosterone/6β-hydroxytestosterone (Fig. 6c) were consistent with their log *P* values. Although the metabolites are generally more hydrophilic than their parent compounds, as indicated by their respective log *P* values, *S*-mephenytoin eluted earlier than 4′-hydroxymephenytoin at both column temperatures (Fig. 6b). This likely occurs because of the difference in the ratio of ionization at pH 4.8 (mobile phase); thus, it is considered that *S*-mephenytoin was more ionized than 4′-hydroxymephenytoin at this pH value.

**Figure 7:**
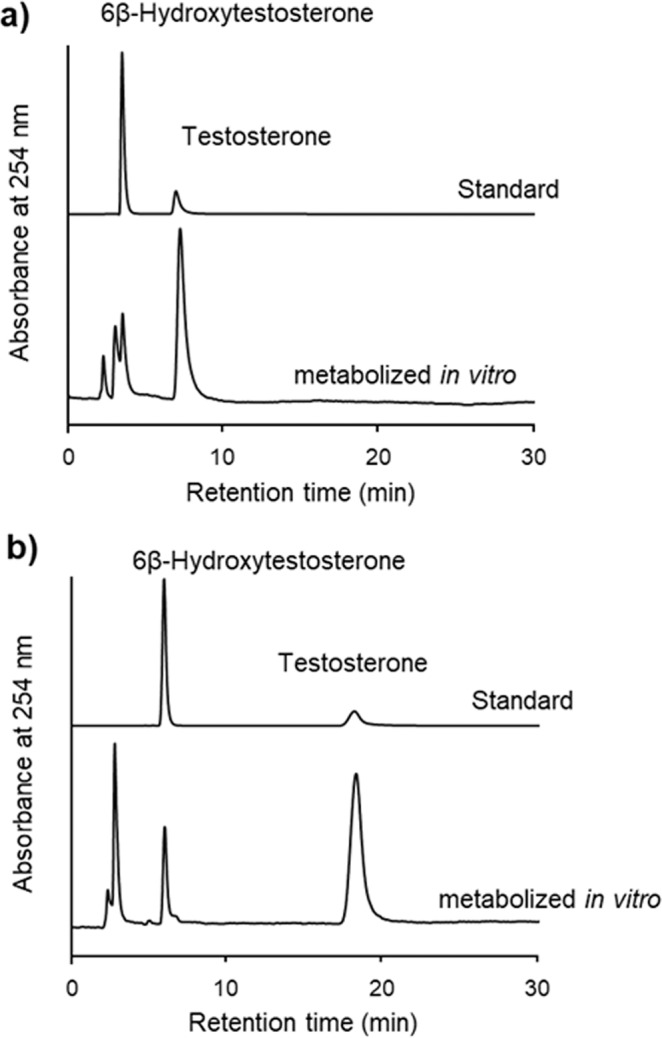
Effect of temperature on the retention times of testosterone and 6β-hydroxytestosterone at 10 °C (**a**) and 40 °C (**b**).

The results of the *in vitro* metabolism test using human CYP3A4 with testosterone and its metabolite are shown in Fig. 7. As seen, the peaks of both testosterone and its metabolite overlap with impurities derived from human CYP3A4 or other materials at a column temperature of 10 °C (Fig. 7a). However, good separation of the analytes without any overlapping peaks from impurities was observed with only deionized water as mobile phase at a column temperature of 40 °C (Fig. 7b), suggesting that multiple CYP substrates and their metabolites would be simultaneously separated after *in vitro* metabolism test by this analytical method.

### Simultaneous analysis of five CYP substrates for clinical drug interaction studies

**Figure 8:**
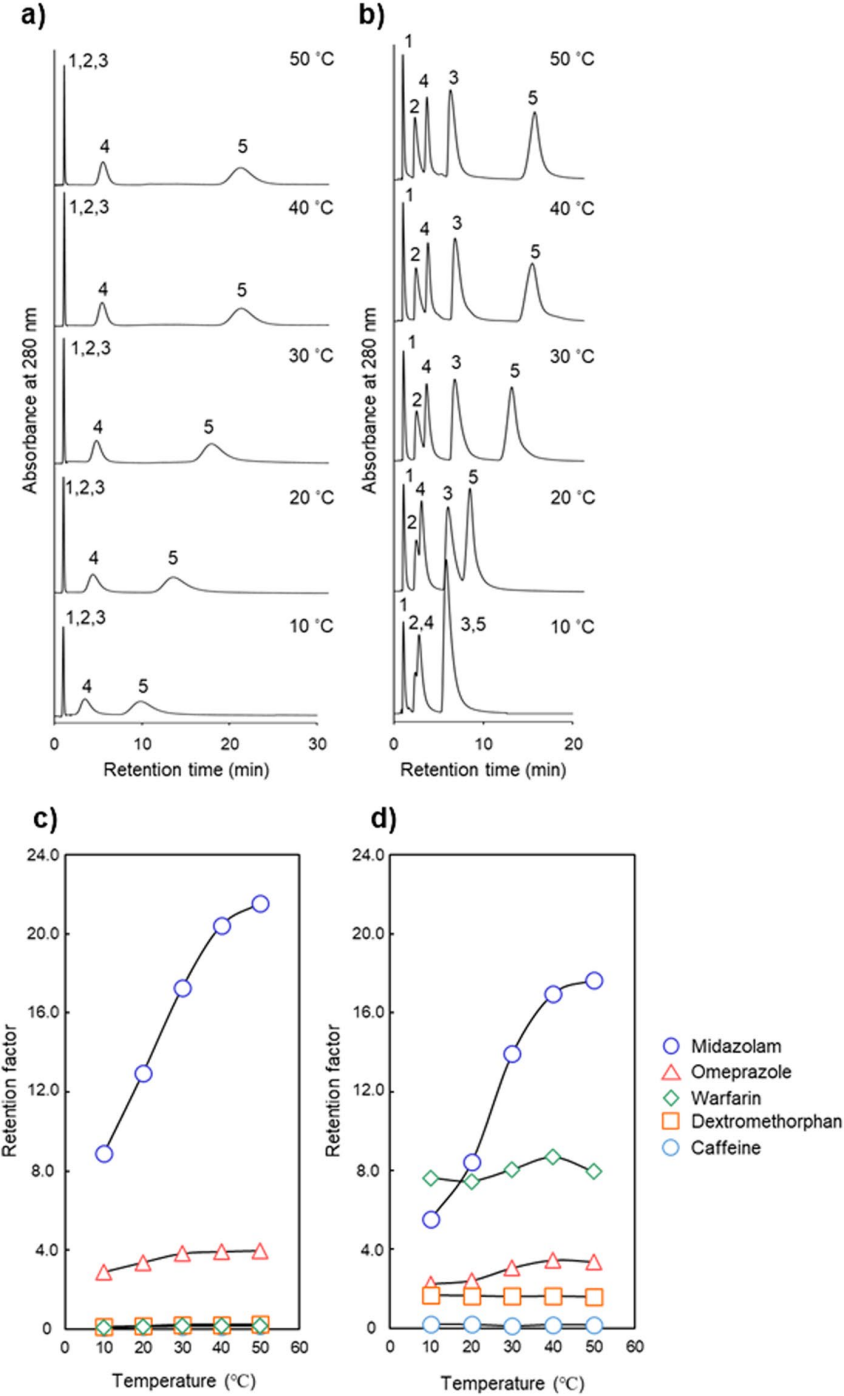
Chromatogram and retention factor of five CYP substrates (1: Caffeine, 2: Dextromethorphan, 3: Warfarin, 4: Omeprazole, 5: Midazolam) at various column temperatures on P(NIPAAm-*co*-BMA) column (**a**) and P(NIPAAm-*co*-L-Trp-OMe) column (**b**); effect of temperature on the retention factor on P(NIPAAm-*co*-BMA) column (**c**) and P(NIPAAm-*co*-L-Trp-OMe) column (**d**).

Five CYP substrates (caffeine for CYP1A2, warfarin for CYP2C9, omeprazole for CYP2C19, dextromethorphan for CYP2D6, and midazolam for CYP3A4; Fig. 3c) were chosen from typical CYP substrates indicated in the FDA guide for clinical drug interaction studies^46^. This cocktail of CYP substrates is also known as the Cooperstown 5 + 1 cocktail^46,49^. These drugs were analyzed using the P(NIPAAm-*co*-BMA) and P(NIPAAm-*co*-L-Trp-OMe) columns at column temperatures from 10 °C to 50 °C with an aqueous mobile phase, 30 mM ammonium acetate buffer at pH 5.8 (Fig. 8a,b). Figure 8c,d show the retention factors at each column temperature for both columns. In the analysis using the P(NIPAAm-*co*-BMA) column, the peaks corresponding to caffeine, dextromethorphan and warfarin overlapped at each temperature (Fig. 8a,c). With the P(NIPAAm-*co*-L-Trp-OMe) column, however, each analyte peak was better separated at each column temperature compared with the P(NIPAAm-*co*-BMA) column and the good separation was shown at 50 °C column temperature (Fig. 8b,d). The separation of the five analytes with the P(NIPAAm-*co*-L-Trp-OMe) column can be attributed to the strong molecular recognition of L-Trp-OMe. We previously reported that introducing a *co*-monomer with an aromatic ring, such as L-phenylalanine, into the polymer unit would enhance molecular recognition viz. NH–π and π–π interactions^21–23^. In contrast to BMA which has no aromatic ring, L-tryptophan has an aromatic indole ring in its structure and has been referred to as the best electron donor among aromatic amino acids like phenylalanine, tyrosine, and histidine^50^. Mikuma *et al*. reported that analytes could easily interact with the NH groups or aromatic rings of P(NIPAAm-*co*-L-Phe-OMe) column at temperatures below the LCST^23^. Even so, our results suggested that their interactions would occur strongly even above the LCST. This difference should arise from the intensity of electron donation between L-Phe-OMe and L-Trp-OMe; therefore, a stronger electron donor of L-Trp-OMe than L-Phe-OMe and the hydrophobic interaction above the LCST would attribute to these results. Furthermore, as these interactions alter depending on the structure of each analyte, it is obvious that analysis of different analytes result in different results. Supplemental data shows the repeatability of the simultaneous analysis with P(NIPAAm-*co*-L-Trp-OMe) column at 50 °C (shown in Table. S1 in supplemental information).

Accordingly, these results indicated that the PNIPAAm-based temperature-responsive column can effectively separate multiple analytes having greatly different structures and properties with only aqueous solvents and without using gradient methods.

## Conclusion

This is the first to report that multiple CYP substrates having greatly different structure and physicochemical properties were well separated using PNIPAAm-based temperature-responsive chromatography with only an isocratic aqueous solvent. In this study, each experiment showed good separation of multiple CYP substrates typically used for *in vitro* or clinical studies without using organic solvents and gradient methods. The aspect to use only isocratic aqueous solvents is efficient because it can significantly reduce the time required to prepare mobile phases, eliminate the need to optimize mobile phase gradient methods, and require less skilled personnel than conventional RPLC. Especially, using only aqueous solvents is advantageous in terms of maintaining biological activity and environmental protection.

In addition, this method is highly versatile because molecular recognition can be arranged by introducing different *co*-monomers into the polymer unit, suggesting that it can potentially apply for the analyses of amino acids, peptides, antibodies, nucleic acids and other bioactive molecules. Several studies have reported that the large amounts of waste produced by medical institutions contribute greenhouse gases^51,52^. When utilizing this current chromatography in medical institutions, the amount of waste from medical institutions should be reduced, which may contribute to environmental protection as well.

Overall, PNIPAAm-based temperature-responsive chromatography represents an attractive simultaneous analytical method for multiple CYP substrates and environment as well. Considering the recent emphasis on environmental protection, the analytical method using only aqueous solvents would be better one and become a breakthrough analytical method in the future.

## Materials and methods

All chemicals used in this study were shown in supplementary information.

### Synthesis of the temperature-responsive polymer and preparation of polymer-grafted silica

P(NIPAAm-*co*-BMA) and P(NIPAAm-*co*-L-Trp-OMe) were synthesized via radical polymerization using 2,2′-azobis(isobutyronitrile) (AIBN) as radical initiator and MPA as chain transfer agent, and the polymer-grafted silica beads were prepared through succinylation and endcapping modifications, as previously reported^23,24,44^. *N*-isopropylacrylamide (NIPAAm) (159 mmol) and BMA (8.40 mmol) or L-Trp-OMe (8.40 mmol) were first dissolved in *N,N*-dimethylformamide (45 mL), followed by the addition of AIBN (0.706 mmol) and MPA (4.13 mmol) into the solution. The mixed solution was stirred at 70 °C after degassed by freeze-pump-thaw cycles. After 5 h reaction, the solution was dropped into diethyl ether to precipitate the polymer. To further purify the polymer, the polymer precipitated in diethyl ether was dissolved into acetone and repeatedly precipitated into diethyl ether. After multiple precipitation cycles the polymer was dried to get a white solid. The molar ratio of NIPAAm with BMA or L-Trp-OMe was at 95:5, respectively.

*N,N*′-dicyclohexylcarbodiimide (0.51 g) and *N*-hydroxysuccinimide (0.29 g) were added to ethyl acetate solution (50 mL) which P(NIPAAm-*co*-BMA) (6.5 g) or P(NIPAAm-*co*-L-Trp-OMe) (6.5 g) was previously dissolved in. The mixed solution was stirred at 4 °C for 2 h, followed by stirred at 25 °C for 24 h. After removing the precipitated dicyclohexylurea by filtration, the filtrate was dropped into diethyl ether for precipitation of the polymer. The residue containing the activated terminal carboxylic acid (1.5 g) was dissolved in 1,4-dioxane (50 mL). After adding aminopropyl silica (3.0 g) to the solution, it was shaken at 25 °C for 24 h. Then, after repetition of this process three times, the resulting polymer-grafted silica beads were washed with methanol (1000 mL) and dried in a vacuum environment. After drying, the beads were suspended in 1,4-dioxane (10 mL) with glycidol (15 mL). The mixed solution was shaken at 25 °C for 24 h to initiate endcapping of the silica amino groups. The resulting silica beads were washed with methanol (150 mL) and dried under vacuum.

### Measuring the LCSTs of the temperature-responsive polymers

The LCST of each polymer was assessed by measuring the optical transmittance of each polymer dissolved in deionized water (5 mg mL^−1^) at the detection wavelength of 500 nm with an UV-Vis spectrophotometer (V-630, JASCO, Tokyo, Japan). PT-31 Peltier system (Krüss, Hamburg, Germany) and ETC-717 controller (JASCO) were used to control the temperature at a heating rate of 0.1 °C min^−1^. The LCST of each polymer was defined as the temperature at 50% of optical transmittance.

### Temperature-responsive chromatography

#### Simultaneous analysis of six CYP substrates for *in vitro* drug interaction assay

A stainless column (150 mm length × 4.6 mm i.d.) was used after packing with P(NIPAAm-*co*-BMA)-grafted silica beads. HPLC system was performed on HITACHI Model L-7100 pump, L-7400 UV detector, D-7500 integrator, and AO-30C column oven (Shodex). The detection wavelength was 220 nm. The mobile phase was 0.1 M ammonium acetate buffer at pH 4.8 at 1.0 mL min^−1^ as flow rate. All drugs were dissolved in tetrahydrofuran at concentrations of 0.25 mg mL^−1^ for use as stock solutions. A mixed sample of phenacetin, coumarin, tolbutamide, *S*-mephenytoin, chlorzoxazone and testosterone was prepared in a ratio of 1:1.5:1:3:1.5:2, respectively. The retention factors of each analyte were assessed using the following equation: retention factor = (*t*_R_ − *t*_0_)/*t*_0_, where *t*_0_ and *t*_R_ are the retention times of uracil and the target analyte, respectively. This mixed sample was also analyzed after spiked in human blood serum. Human serum was obtained from Nissui Pharmaceutical (Tokyo, Japan) and purified using a Sep-pak Plus C18 cartridge (Waters, Milford, MA, US) preconditioned by methanol/deionized water (50:50) and 0.1 M ammonium acetate and eluent (methanol after 0.1 M ammonium acetate). The eluate was evaporated and then dissolved in tetrahydrofuran (1 mL) to obtain blank. The mixed sample 300 μL was mixed in the blank 200 μL as mixture sample spiked in biological fluid.

#### Separation of CYP substrates and metabolites by temperature-responsive chromatography

A stainless column (150 mm length × 4.6 mm i.d.) packed with P(NIPAAm-*co*-BMA)-grafted silica beads was used. HPLC system was performed on those same as the first analysis. The detection wavelength was 220 nm for the analyses of phenacetin/acetaminophen and *S*-mephenytoin/4′-hydroxymephenytoin and 254 nm for the analysis of testosterone/6β-hydroxytestosterone. The mobile phase for the analyses of phenacetin/acetaminophen and *S*-mephenytoin/4′-hydroxymephenytoin was 0.1 M ammonium acetate buffer at pH 4.8 at a flow rate of 1.0 mL min^−1^. For the analysis of testosterone/6β-hydroxytestosterone, the mobile phase was deionized water at a flow rate of 1.0 mL min^−1^. Phenacetin and acetaminophen were dissolved in the mobile phase at concentrations of 0.2 mg mL^−1^ for use as stock solutions, and their samples were mixed at a ratio of 1:1. *S*-Mephenytoin and 4′-hydroxymephenytoin were dissolved in the mobile phase at a concentration of 0.16 mg mL^−1^ for use as stock solutions, and their samples were mixed at a ratio of 3:2. For use as stock solutions, testosterone was dissolved in deionized water at a concentration of 25 μg mL^−1^ and 6β-hydroxytestosterone was dissolved in a 50% acetonitrile solution at a concentration of 10 mg mL^−1^. A sample of testosterone and 6β-hydroxytestosterone was mixed at a ratio of 66:1.

#### Analysis of testosterone/6β-hydroxytestosterone *in vitro* metabolism tests

P(NIPAAm-*co*-BMA)-grafted silica beads were packed into a stainless column (150 mm length × 4.6 mm i.d.) that was connected to HPLC system (HITACHI Model L-7100 pump, L-7405 UV detector, D-7100 integrator) with an AO-30C column oven (Shodex). The detection wavelength was 254 nm. The mobile phase was deionized water at a flow rate of 1.0 mL min^−1^. Testosterone (1.0 mg mL^−1^ in methanol, 5 μL), CYP3A4 (20 pmol, 10 μL, Corning), NADPH Regenerating System Solution A (25 µL, Corning) and NADPH Regenerating System Solution B (5 µL, Corning) were added in phosphate buffer (pH 7.4, 445 µL). After incubation at 37 °C for 10 min, acetonitrile (250 µL) was added. Thereafter, the solution was centrifuged at 3000 rpm for 10 min. The collected supernatant was evaporated and dissolved in deionized water (1 mL) and then purified using a Sep-pak Plus C18 cartridge and eluent (methanol/deionized water (60:40)). The eluate was again evaporated and then dissolved in deionized water (1 mL) to obtain a sample solution.

#### Simultaneous analysis of five CYP substrates for clinical drug interaction studies

A stainless column (50 mm length × 2.1 mm i.d.) packed with P(NIPAAm-*co*-BMA)- or P(NIPAAm-*co*-L-Trp-OMe)-grafted silica beads was used and connected to ultra HPLC system (HITACHI ChromasterUltra Rs, 6170 pump, 6270 autosampler, 6310 column oven, 6430 diode array detector). The detection wavelength was 280 nm. The mobile phase was 30 mM ammonium acetate buffer at pH 5.8 at 0.2 mL min^−1^ as flow rate. All drugs were dissolved in tetrahydrofuran at concentrations of 1.0 mg mL^−1^ for use as stock solutions. A mixed sample of caffeine, warfarin, omeprazole, dextromethorphan and midazolam was prepared in a ratio of 1:8:6:20:25, respectively. The same equation as described before was used for calculation of the retention factors of each analyte.

## Supplementary information

Supplementary information.
