# Supplementary material for: Green analytical method for the simultaneous analysis of cytochrome P450 probe substrates by poly(N-isopropylacrylamide)-based temperature-responsive chromatography
Source: Sci Rep. 2020 Jun 1;10:8828. doi: 10.1038/s41598-020-65270-z (PMC7264224; doi:10.1038/s41598-020-65270-z)
Supplement: Supplementary file 1 — Supplementary information. [file 41598_2020_65270_MOESM1_ESM.pdf]

Green analytical method for the simultaneous analysis of cytochrome P450 probe substrates by poly(*N*-isopropylacrylamide)-based temperature-responsive chromatography

Yutaro Maekawa, Naoya Okamoto, Yuji Okada, Kenichi Nagase, Hideko Kanazawa*
Faculty of Pharmacy, Keio University, 1-5-30, Shibakoen, Minato-ku, Tokyo 105-8512, Japan
*Corresponding author: Hideko Kanazawa, Tel: +81-3-5400-2657, Fax: +81-3-5400-1378, E-mail: kanazawa-hd@pha.keio.ac.jp

Table. S1. Repeatability of the present simultaneous analysis for multiple CYP substrates; relative standard deviation (RSD) was calculated from the data (mean and standard deviation (SD)) of triplicate sample (n=3) at a given concentration of each analyte: RSD was calculated from the data (mean and SD) of triplicate sample at a given concentration of each analyte.

Analyte	Concentration	Retention time (min)			Area		
	(mg mL^{-1})	Mean	(SD)	RSD	Mean	(SD)	RSD
Caffeine	0.00625	1.05	(0.000577)	0.000551	181000	(7020)	0.0387
	0.0125	1.05	(0.000577)	0.000552	364000	(7830)	0.0215
	0.025	1.04	(0.00116)	0.00111	744000	(15400)	0.0207
Dextro-methorphan	0.125	2.51	(0.00423)	0.00168	765000	(37300)	0.0488
	0.25	2.47	(0.00200)	0.000810	1680000	(8850)	0.00527
	0.5	2.44	(0.000681)	0.000279	3320000	(30500)	0.00918
Warfarin	0.05	8.24	(0.000577)	0.0000700	1460000	(33100)	0.0226
	0.1	8.17	(0.000850)	0.000104	2970000	(47600)	0.0160
	0.2	8.11	(0.00565)	0.000696	6050000	(47700)	0.00789
Omeprazole	0.0375	4.03	(0.00869)	0.00215	1180000	(32100)	0.0272
	0.075	4.02	(0.00243)	0.000603	2430000	(42100)	0.0173
	0.15	3.99	(0.00637)	0.00160	4740000	(46100)	0.00972
Midazolam	0.15	16.4	(0.00155)	0.0000946	4760000	(41000)	0.00860
	0.3	16.4	(0.0127)	0.000775	9600000	(51100)	0.00532
	0.6	16.5	(0.00265)	0.000161	19700000	(121000)	0.00612

Supplementary Materials and methods

Chemicals

N-Isopropylacrylamide (NIPAAm) was kindly provided by KJ Chemicals (Tokyo, Japan) and recrystallized with *n*-hexane for its purification. BMA was purchased from Wako Pure Chemical Industries (Osaka, Japan). L-Trp-OMe was prepared from L-tryptophan, which was obtained

from the Peptide Institute (Osaka, Japan), through methyl esterification of L-tryptophan and reaction with acryloyl chloride and the methyl ester in the presence of trimethylamine using previously reported methods [1-3]. 2,2'-Azobis(isobutyronitrile) and 1,4-dioxane were obtained from Wako Pure Chemical Industries. *N,N*-dimethylformamide, 3-Mercaptopropionic acid and *N,N'*-dicyclohexylcarbodiimide were obtained from Kanto Chemicals (Tokyo, Japan). *N*-Hydroxysuccinimide was obtained from Merck Japan (Tokyo, Japan). Aminopropyl silica (average diameter 5 μm, pore size 12 nm) was obtained from Nishio Kogyo (Tokyo, Japan) and YMC (Kyoto, Japan). Deionized water purified by a Purelite PRB (Organo, Tokyo, Japan) was used to prepare the eluent and samples. The other chemicals used in this study were of analytical reagent grade.

Supplementary References

1. Li, K., Tan, G., Huang, J., Song, F., You, J. Iron-catalyzed oxidative C-H/C-H cross-coupling: an efficient route to alpha-quaternary alpha-amino acid derivatives. *Angew Chem Int Ed Engl.* **52**, 12942-12945 (2013).
2. Sanda, F., Abe, T., Endo, T. Syntheses and radical polymerizations of optically active (meth)acrylamides having amino acid moieties. *J Polym Sci A Polym Chem.* **35**, 2619-2629 (1997).
3. Moore, B. L., O'Reilly, R. K. Preparation of chiral amino acid materials and the study of their interactions with 1,1-Bi-2-naphthol. *J Polym Sci A Polym Chem.* **50**, 3567-3574 (2012).
